# MLN64 Transport to the Late Endosome Is Regulated by Binding to 14-3-3 *via* a Non-canonical Binding Site

**DOI:** 10.1371/journal.pone.0034424

**Published:** 2012-04-13

**Authors:** Anastasia Liapis, Fannie W. Chen, Joanna P. Davies, Rong Wang, Yiannis A. Ioannou

**Affiliations:** Department of Genetics & Genomic Sciences, The Mount Sinai School of Medicine, New York, New York, United States of America; Institute of Molecular and Cell Biology, Singapore

## Abstract

MLN64 is an integral membrane protein localized to the late endosome and plasma membrane that is thought to function as a mediator of cholesterol transport from endosomal membranes to the plasma membrane and/or mitochondria. The protein consists of two distinct domains: an N-terminal membrane-spanning domain that shares homology with the MENTHO protein and a C-terminal steroidogenic acute regulatory protein (StAR)-related lipid transfer (START) domain that binds cholesterol. To further characterize the MLN64 protein, full-length and truncated proteins were overexpressed in cells and the effects on MLN64 trafficking and endosomal morphology were observed. To gain insight into MLN64 function, affinity chromatography and mass spectrometric techniques were used to identify potential MLN64 interacting partners. Of the 15 candidate proteins identified, 14-3-3 was chosen for further characterization. We show that MLN64 interacts with 14-3-3 *in vitro* as well as *in vivo* and that the strength of the interaction is dependent on the 14-3-3 isoform. Furthermore, blocking the interaction through the use of a 14-3-3 antagonist or MLN64 mutagenesis delays the trafficking of MLN64 to the late endosome and also results in the dispersal of endocytic vesicles to the cell periphery. Taken together, these studies have determined that MLN64 is a novel 14-3-3 binding protein and indicate that 14-3-3 plays a role in the endosomal trafficking of MLN64. Furthermore, these studies suggest that 14-3-3 may be the link by which MLN64 exerts its effects on the actin-mediated endosome dynamics.

## Introduction

MLN64 is an integral membrane protein localized primarily in the late endosome [1], [2]. Its N-terminus is homologous to another late endosomal protein of unknown function called MENTHO, with which it may interact [3], [4]. Cross-linking studies indicate that the MLN64 N-terminal domain may also bind cholesterol [4], whereas overexpression of MLN64 leads to cholesterol accumulation in the endosomal/lysosomal (E/L) compartment of COS1 cells, similar to depletion of NPC1 [5].

A role for MLN64 in cholesterol trafficking was first proposed based upon its sequence homology to the functional region of the steroidogenic acute regulatory (StAR) protein [2], [6]. X-ray crystallographic analysis of the START domain revealed the existence of a hydrophobic cavity that could potentially accommodate a single cholesterol molecule [7], supporting the idea that MLN64 could function as a cholesterol transporter. However, although the START domain of MLN64 can promote cholesterol transport *in vitro* [8], targeted mutation of this domain does not affect cholesterol metabolism in mice [9]. Although it is possible that this result can be explained by species differences or the lack of an MLN64 null mouse model, more questions are raised when considering that in the mouse MLN64 was found to be largely absent from steroidogenic cells [10].

To further investigate the function of MLN64 and its lipid binding domains, different portions of the protein were overexpressed in mammalian cells and their effect on endosomal and plasma membrane cholesterol levels as well as cholesterol transport from the E/L system was examined. To further characterize the function of MLN64, we identified candidate interacting partners through a combination of affinity purification and mass spectrometry and verified that MLN64 is a novel 14-3-3 interacting protein. Finally, we examined the effect of 14-3-3 binding on MLN64-mediated cholesterol trafficking as well as the transport of MLN64 to the late endosome.

## Results

### Effects of MLN64 Overexpression

**Figure 1:**
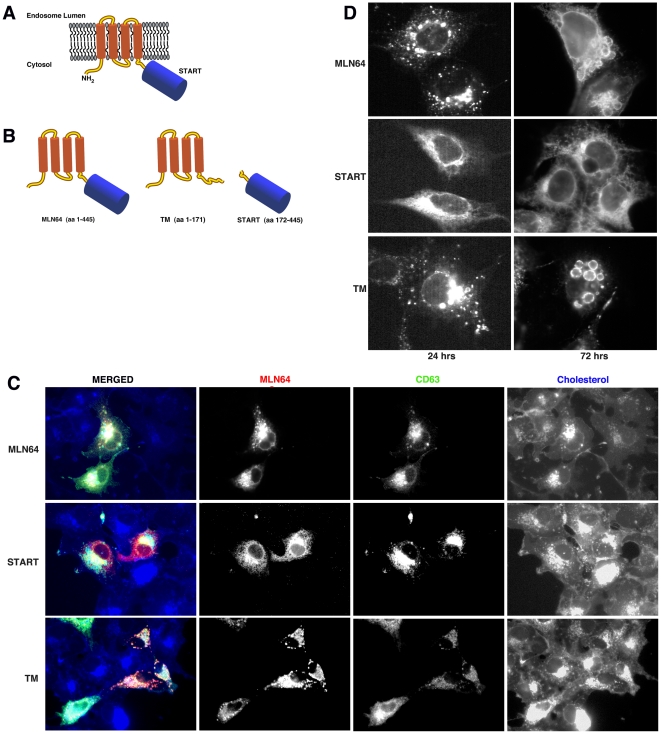
Expression studies of full length and truncated MLN64 protein. A) MLN64 is a late endosomal protein that consists of four transmembrane segments and a cytosolic START domain. B) Schematic of full length and truncated MLN64 proteins containing only the transmembrane domains (TM) or the cytosolic START domain (START). C) Expression of MLN64, TM or START (red signal) causes accumulation of free cholesterol (blue signal) in the E/L system but only MLN64 and TM colocalize in late endosomes with CD63-YFP (green signal), whereas START displays an expression pattern characteristic of the ER. D) Cells expressing MLN64 and TM, but not START, display enlarged endosomal vesicles at 24 hours post-transfection, and this phenotype becomes more pronounced after 72 hours.

The MLN64 protein is composed of two distinct domains, the N-terminal transmembrane domain, which has been shown to bind cholesterol in the membrane [4] and the C-terminal cytosolic START domain, which has been shown to mobilize cholesterol *in vitro* [8] (Figure 1A). To evaluate the effect of MLN64 overexpression on cholesterol trafficking, constructs were generated encoding full-length MLN64, its START domain (START), and the transmembrane domains (TM) (Figure 1B).

COS7 cells were cotransfected with the Flag-tagged MLN64 constructs and a CD63-YFP fusion construct to label the late endosomes. Similar to previous results [1], MLN64, TM or START overexpressing cells displayed cholesterol-filipin staining at higher levels than untransfected cells. However, only the full-length MLN64 and TM constructs colocalized in late endosomes with CD63, whereas the START fragment displayed an expression pattern characteristic of the endoplasmic reticulum (ER) (Figure 1C), suggesting that endosomal targeting of the protein requires the transmembrane domains of MLN64. No further studies were carried out using the START domain due to it’s inability to target to vesicles.

In order to characterize the effect of MLN64 overexpression further, the morphology of late endocytic organelles was examined in cells expressing full-length MLN64 or the truncated constructs for an extended period. At 24 hours, the MLN64 and TM constructs localized to slightly enlarged endosomal vesicles (Figure 1D), while the START domain displayed staining characteristic of the ER as discussed above. However, at 72 hours after transfection, the effect of full length MLN64 and TM overexpression was grossly exacerbated and endocytic vesicles appeared even further enlarged. The largest vesicles were found in the TM-expressing cells (Figure 1D), suggesting a function for the transmembrane portion of the protein in membrane dynamics.

To determine if MLN64 overexpression could affect LDL-cholesterol transport to the ER for esterification, esterification assays were performed in cells transfected with each of the three MLN64 constructs. MLN64 overexpression did not cause a significant change in esterification rates compared with untransfected cells (data not shown), suggesting that MLN64 is not directly involved in the delivery of LDL-cholesterol to ACAT in the ER.

### Identification of Candidate MLN64-interacting Proteins

**Figure 2:**
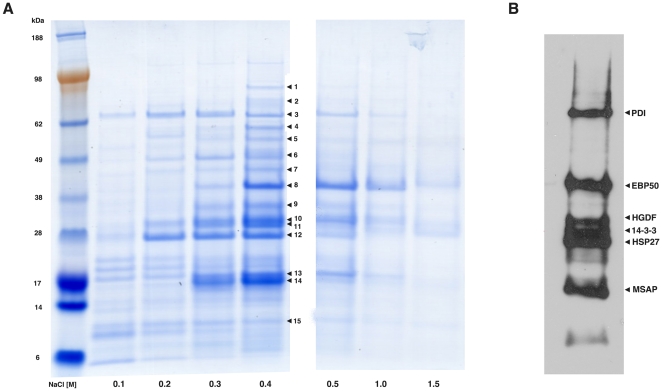
Identification of MLN64-interacting proteins. A) Proteins bound to a START-affinity column were eluted with 0.1–1.5 M NaCl and resolved by gel electrophoresis. Proteins that remained associated at 0.4 M NaCl were isolated from the gel and identified by mass spectrometry. B) The 0.4 M NaCl elution fraction was also subjected to a blot overlay experiment using recombinant START protein to confirm potential binding partners.

To probe further the function of MLN64 and its role in lipid transport, the START domain of MLN64 was used for affinity chromatography of human MCF7 cell extracts to isolate cytosolic MLN64-interacting proteins. The START affinity column was eluted with increasing salt concentrations and the resulting fractions were separated by gel electrophoresis (Figure 2A). A total of 15 proteins remained associated with START at high salt concentrations and were excised from the gel and identified by tandem mass spectrometry. Several of the identified proteins were unknown while a number of proteins are known to reside in cellular compartments that are distinct from the cytoplasmic location of the MLN64 START domain (Table 1).

**Table 1:** MLN64-interacting proteins identified by mass spectrometry.

Band	Protein	Identifier
1	Heat shock protein HSP 90-alpha (HSP 86)	GI: 14124932
2	Protein disulfide isomerase (PDI)	GI: 4758304
3	Stress-induced phosphoprotein 1 (Hsp70/Hsp90-organizing protein)	GI: 5803181
4	CAA28775.1 Hypothetical protein	GI: 35655
5	Calreticulin	GI: 4757900
6	Solute carrier family 9, sodium/hydrogen exchanger (EBP50)	GI: 4759140
7	AAH17450 Unknown	GI: 16924319
8	Hepatoma-derived growth factor (HDGF)	GI: 4758516
9	Heat shock protein 27 (HSP27)	GI: 662841
10	14-3-3 Protein tau	GI: 1526541
11	Tumor protein D52	GI: 4827038
12	MIR-interacting saposin-like protein (MSAP)	GI: 7657176
13	Chromatin modifying protein 4a (CHMP4)	GI: 315259102
14	Eukaryotic translation initiation factor 5A-1 (eIF5AI)	GI: 4503545
15	Mitochondrial import inner membrane translocase subunit (Tim8A)	GI: 7305577

Thus, to further confirm and limit the number of potential binding partners, a blot overlay experiment using recombinant START protein was performed (Figure 2B). Six proteins bound recombinant START on this overlay, including protein disulfide-isomerase (PDI), sodium/hydrogen exchanger 3 (EBP50), hepatoma-derived growth factor (HDGF), heat shock protein 27 (Hsp27), 14-3-3, and MIR-interacting saposin-like protein (MSAP). Of these six candidates, 14-3-3 was selected first for further analysis.

### MLN64 is a Novel 14-3-3 Binding Protein

**Figure 3:**
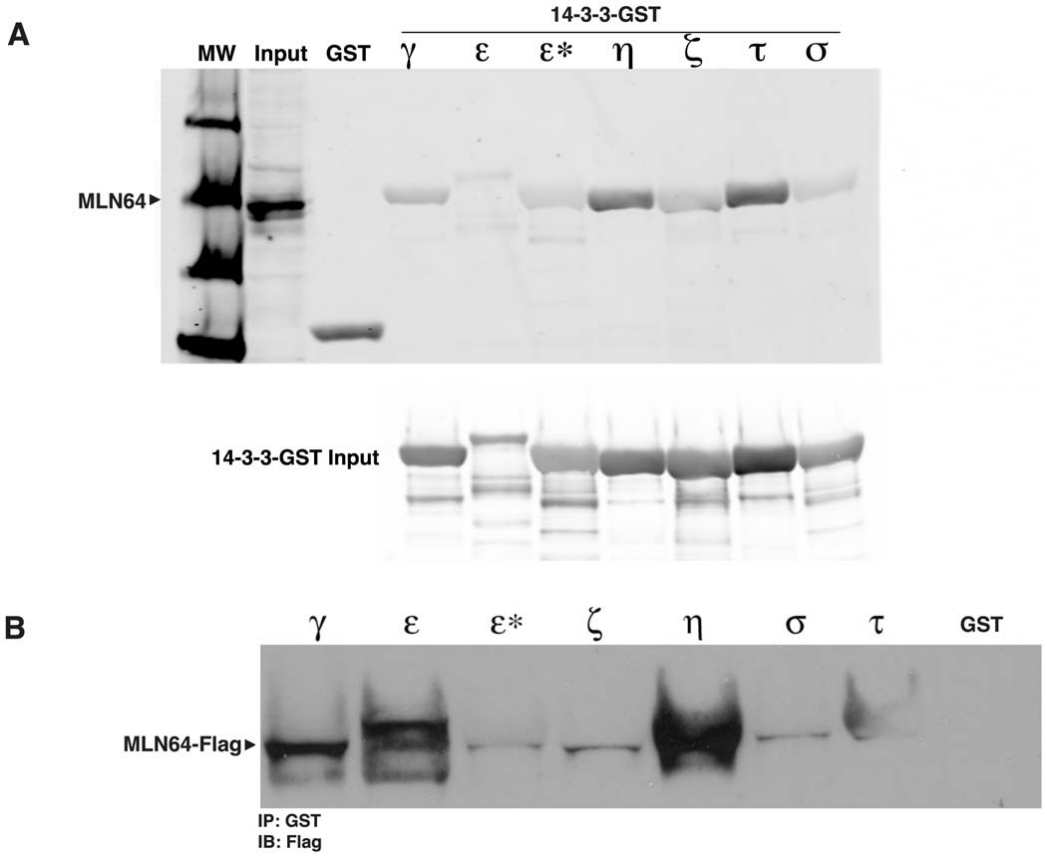
MLN64 interacts with 14-3-3 *in vitro*. A) Western blot analysis of endogenous MLN64 from cell lysates bound to purified GST-tagged 14-3-3 isoforms indicates interactions between MLN64 and 14-3-3 γ, ε, η, ζ, τ and σ isoforms. ε* denotes a 14-3-3 mutant that cannot bind target proteins. B) Western blot analysis of purified Flag-tagged MLN64 bound to purified 14-3-3-GST isoforms indicates the strongest interactions with the 14-3-3 η and τ isoforms.

Since affinity purification and blot overlay showed that 14-3-3 can interact with the recombinant START domain of MLN64, its interaction with full-length MLN64 was further investigated. Bacterially expressed glutathione-S-transferase (GST)-tagged 14-3-3 isoforms were used to pull down endogenous MLN64 from an Huh7 cell lysate. Western blot analysis of MLN64 bound to 14-3-3-GST beads showed that MLN64 can interact with most 14-3-3 isoforms, including the γ, ζ, η, and τ isoforms, with the strongest binding shown by the η and τ isoforms (Figure 3A). As an internal control, a mutant 14-3-3 ε isoform (ε*) that cannot bind target proteins [11] did not show any interaction with MLN64.

To determine whether MLN64 interacts preferentially with a specific 14-3-3 isoform, equal amounts of MLN64-Flag-expressing Huh7 cell lysates were incubated with each GST-14-3-3 isoform and immunoprecipitated with anti-GST antibody. Western blot analysis revealed that MLN64 bound most strongly to the 14-3-3 η isoform, less tightly to β and γ isoforms, and weakly if at all to the ε* mutant, σ and ζ isoforms (Figure 3B). These results varied slightly from the results using endogenous MLN64 (Figure 3A), however the η and τ isoforms consistently bound very strongly to MLN64, while the ε* mutant and GST controls did not, indicating a direct, strong interaction of MLN64 to 14-3-3.

### Endogenous MLN64 Specifically Interacts with 14-3-3 In vivo

**Figure 4:**
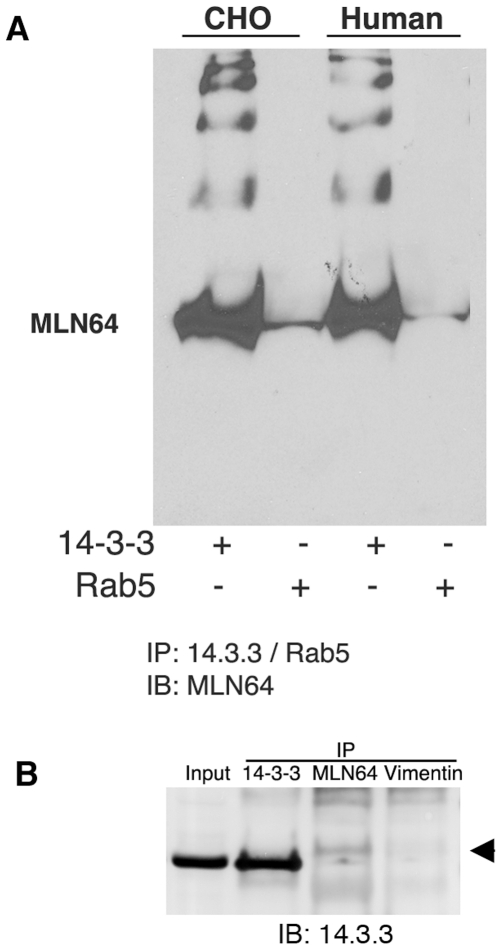
MLN64 interacts with 14-3-3 *in vivo*. A) Western blot analysis of endogenous MLN64 from cell lysates immunoprecipitated with an antibody that recognizes all seven 14-3-3 isoforms indicates that MLN64 and 14-3-3 interact *in vivo*. The small amount of protein precipitated with the Rab5 antibody most likely represents a population in transit to the late endosome. B) Human cell extracts were immunoprecipitated using anti-14-3-3, anti-MLN64 or anti-vimentin antibodies. Precipitates were analyzed by Western blot probed for the presence of 14-3-3 proteins.

To address the *in vivo* relevance of the MLN64-14-3-3 interaction, co-immunoprecipitations of endogenous proteins were performed using CHO and MCF7 cell lysates. Endogenous MLN64 protein was precipitated using a 14-3-3 antibody that recognizes all seven 14-3-3 isoforms (14-3-3, H-8). An antibody against Rab5, a GTPase associated with early but not late endosomes, was used as a control. Samples were analyzed by SDS-PAGE and then probed with an anti-MLN64 antibody. As shown in Figure 4, a substantial amount of MLN64 can be precipitated from both lysates with a 14-3-3 antibody but not with the Rab5 antibody. The reverse experiment using the MLN64 antibody to precipitate 14-3-3 failed to detect an interaction between the two proteins (data not shown). This result is most likely due to the fact that the MLN64 antibody, which was raised against the START domain, recognizes the epitope necessary for the specific interaction with 14-3-3 and thus the interaction is blocked (see below). To further confirm these results human cell extracts were immunoprecipitated with antibodies against 14-3-3, MLN64 or vimentin as a control followed by immunoblotting with an anti = 14-3-3 antibody. As shown in Figure 4B immunoprecipitation of endogenous MLN64 coprecipitates 14-3-3 proteins whereas immunoprecipitation with an anti-vimentin antibody, as a control, does not (Figure 4B).

### Generation of an MLN64 Mutant Deficient in 14-3-3 Binding

For many target proteins, 14-3-3 binding occurs on specific phosphorylated serine and threonine residues [12]. Two optimal 14-3-3-binding phosphopeptide motifs, MODE I (RSXpSXP) and MODE II (RXY/TXpSXP), have been isolated in oriented peptide library screens [13]. The MLN64 protein does not contain the canonical sequences reported in many 14-3-3 binding partners, therefore to identify non-canonical binding sites we utilized the MiniMotif Miner application, a database prediction tool that focuses on identifying small subsets of short motifs [14]. This tool was validated by analyzing thousands of confirmed examples of protein motifs as well as by confirming predictions of previously unidentified 14-3-3 motifs in EFF-1, a *Caenorhabditis elegans* protein found to be necessary and sufficient for most somatic cell fusions in development [15].

**Figure 5:**
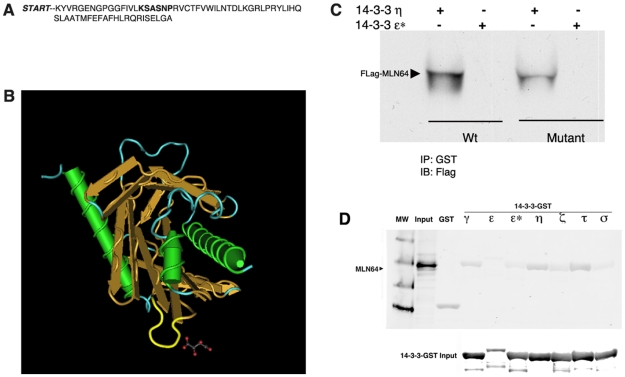
The MLN64 START domain is important for interactions with 14-3-3. A) An atypical 14-3-3 binding site at position 392 of the START domain of MLN64. B) According to a ribbon diagram of the crystal structure of the START domain, the predicted 14-3-3 binding site lies in an exposed loop (yellow) on the surface of the protein. C) Immunoprecipitation and western blot analysis of lysates from cells transfected with either MLN64-Flag (Wt) or MLN64*-Flag (Mut) indicates that the mutant MLN64 protein displays greatly reduced binding to the η isoform relative to the Wt protein. ε* denotes a 14-3-3 isoform that cannot bind target proteins. D) Similar to Figure 3A, Western blot analysis of expressed mutant MLN64*-Flag from cell lysates bound to purified GST-tagged 14-3-3 isoforms indicates little or no interaction of mutant MLN64* and 14-3-3 γ, ε, η, ζ, τ and σ isoforms.

Using MiniMotif Miner, an atypical binding site was identified in the MLN64 protein sequence at position 392 of the START domain (KSASNP) (Figure 5A). This C-terminal site displays a high degree of similarity with the canonical MODE I motif of the consensus binding motifs. To investigate whether this predicted site was indeed required for 14-3-3 binding, a putative binding mutant was generated by alanine substitution. Based on the known crystal structure of the MLN64 START domain, the identified site appears as a small loop on the surface of the protein (Figure 5B). To preserve structural kinks imposed by the proline residue in the START domain, the proline in the last position of the C-terminal motif (KSASNP) was preserved.

To determine whether the mutant MLN64 protein could bind 14-3-3 *in vitro*, GST pulldown experiments were carried out using lysates from Huh7 cells transfected with either MLN64-Flag (Wt) or MLN64*-Flag (Mutant) and the 14-3-3 η isoform that showed the highest degree of binding to Wt MLN64 (Figure 3A). Mutant MLN64 exhibited only weak binding to 14-3-3 η compared to the Wt protein (Figure 5C), strongly suggesting that the mutated site contributes to the interaction between MLN64 and 14-3-3. Furthermore, the low levels of binding to the mutant protein may be due to retention of the terminal proline in the 14-3-3 binding site. As expected, neither the Wt nor mutant MLN64 proteins displayed any binding to the 14-3-3 ε* negative control.

As further support for this conclusion the equal amounts of mutant MLN64*-Flag-expressing Huh7 cell lysates were incubated with each GST-14-3-3 isoform and immunoprecipitated with anti-GST antibody as was shown for Wt MLN64 in Figure 3A above. As shown in Figure 5D there was very little, if any, interaction of mutant MLN64*-Flag with the various 14-3-3 isoforms.

### Mutation of the 14-3-3 Binding Site Delays Trafficking of MLN64 to the Late Endosome 

**Figure 6:**
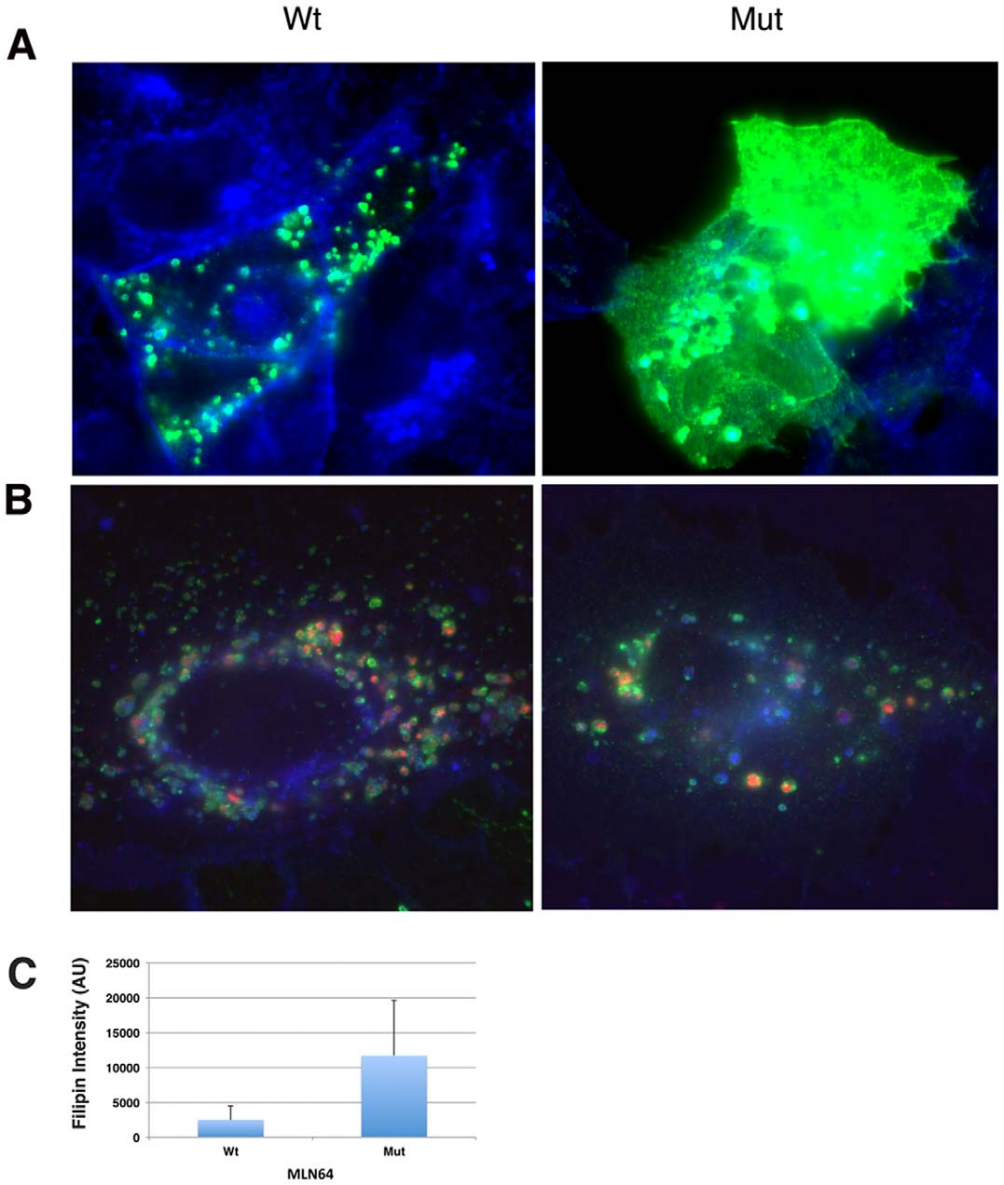
Expression of mutant MLN64 causes aberrant MLN64 and lipid trafficking in cells. A) Expression of either Wt or mutant MLN64 protein (red) in cells results in some co-localization with CD63-YFP (green); however, Wt MLN64 appears primarily vesicular, whereas mutant MLN64 exhibits more plasma membrane staining. Nuclei (blue) were stained with Hoechst 33342. B) Expression of either Wt or mutant MLN64 protein (green) results in accumulation of free cholesterol (blue) and GM1 (red) in endocytic vesicles. Cells transfected with mutant MLN64 accumulated more cholesterol than cells transfected with Wt MLN64; however, the levels of GM1 in both cell types were the equivalent. C) Quantitation of cholesterol accumulation in cells expressing either Wt MLN64 (Wt) or mutant MLN64* (Mut). AU; arbitrary units.

The normal trafficking itinerary of MLN64 in the cell involves traveling first to the plasma membrane before being targeted to the late endocytic compartment [8]. Subcellular localization studies have shown that MLN64 co-localizes partially with markers associated with early, recycling and sorting endosomes but is predominantly associated with late endosomal vesicles [1]. These observations suggest that MLN64 may move dynamically through the endocytic pathway and that disruption of its trafficking may affect its function. To determine if Wt and mutant MLN64 proteins trafficked to the appropriate cellular compartment (late endosome), Flag-tagged MLN64 constructs were co-expressed with a CD63-YFP fusion protein, which serves as a marker for late endosomal vesicles as well as the plasma membrane. Immunofluorescence staining at 24 hours post-transfection revealed largely late-endosomal staining for Wt MLN64 as indicated by co-localization with CD63 (Figure 6A, Wt).

However, some differences in localization patterns became apparent in the 14-3-3 binding mutant of MLN64, which appeared to accumulate at the plasma membrane much more than the Wt MLN64 protein (Figure 6A, Mut). These results are consistent with published data indicating that MLN64 traffics to the plasma membrane prior to reaching its final destination on the late endosomes [8] and furthermore indicates that this trafficking is affected by mutation of the putative C-terminal 14-3-3 binding site of MLN64.

Given the potential importance of MLN64 in cholesterol trafficking we next determined whether overexpression of Wt and mutant MLN64 proteins could cause cholesterol accumulation in the E/L system. Consistent with our previous results (Figure 1C), expression of the Wt and mutant MLN64 proteins caused cholesterol accumulation in the E/L system and furthermore, the mutant MLN64 protein caused a higher degree of accumulation than the Wt protein (Figure 6B, 6C). Cells were also stained with the cholera toxin B subunit to label the monosialotetrahexosyl ganglioside (GM1). Expression of the mutant MLN64 protein did not affect the levels of GM1 in late endocytic vesicles relative to the Wt protein (Figure 6B); however, the amount of GM1 in transfected cells was higher than in the untransfected control (not shown).

**Figure 7:**
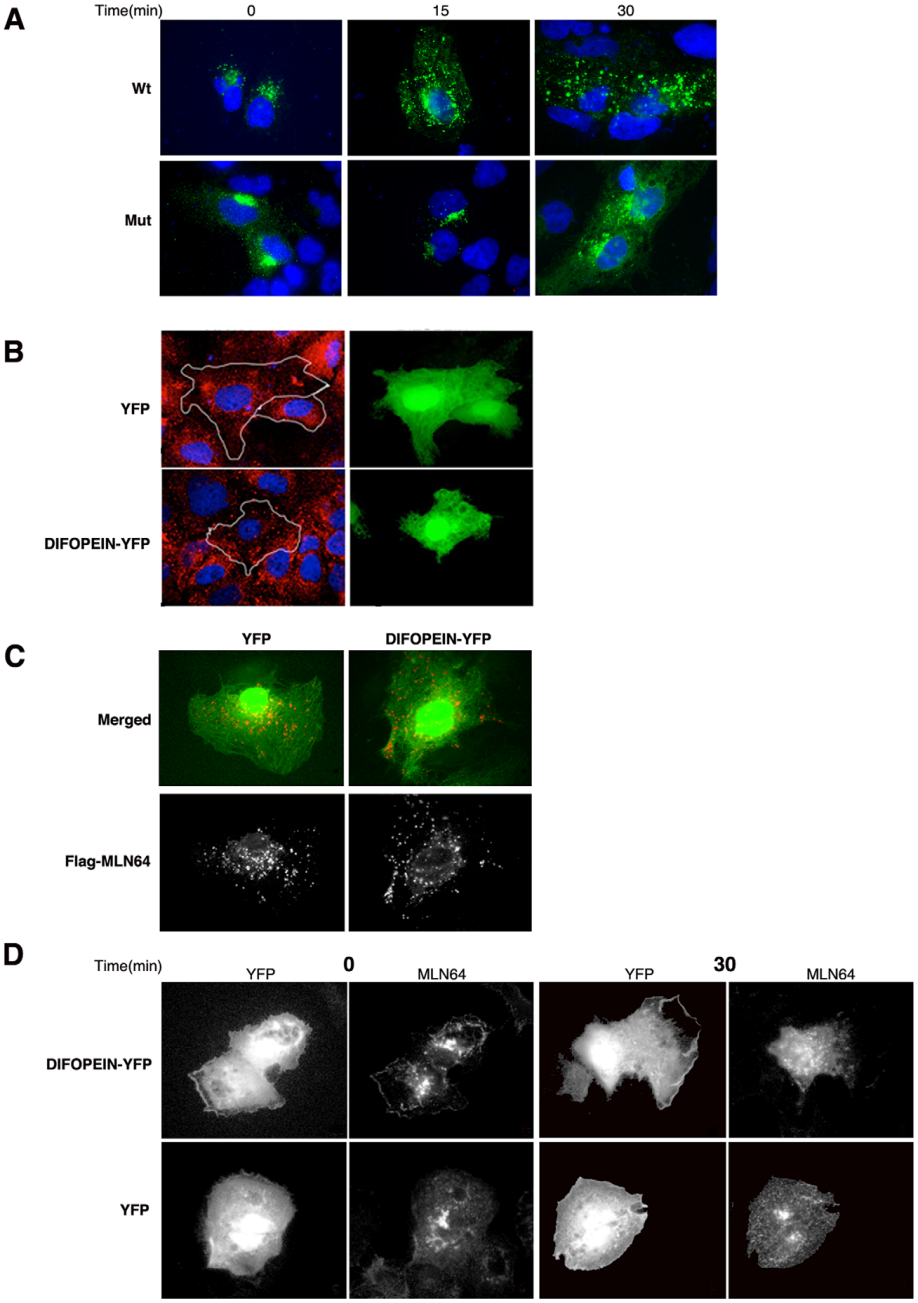
Lack of 14-3-3 binding causes delayed trafficking of MLN64 to the endosomal system. A) Huh7 cells transfected with MLN64-Flag (Wt) or MLN64*-Flag (Mut) for 24 hours were incubated at 20°C for 2 hr to cause accumulation of the protein at the Golgi. Immediately preceding release from the block, MLN64 proteins (green) exhibited a perinuclear staining pattern characteristic of the Golgi (t  =  0). Shortly after release from the block, Wt MLN64 is found in both vesicular compartments and the plasma membrane (t = 15, t = 30), in contrast to mutant MLN64, which remains primarily in the Golgi at 15 minutes. Only a small population of mutant protein appears on the plasma membrane and in vesicles at 30 minutes. Nuclei were counterstained with Hoechst 33342 (blue signal). B) MLN64-positive late endosomes (red) exhibit dissimilar localization in Huh7 cells transfected with DIFOPEIN-YFP or YFP alone (green) for 24 hrs. In YFP-expressing cells, MLN64 exhibits a characteristic perinuclear distribution, whereas in cells expressing DIFOPEIN-YFP, in which 14-3-3-ligand interactions are blocked, MLN64 recedes to the cell periphery. C) Co-expression of YFP (green) and MLN64-Flag (red) in Huh7 cells does not affect the normal perinuclear distribution of endosomes. In contrast, co-expression of DIFOPEIN-YFP with MLN64-Flag causes MLN64-positive vesicles to disperse toward the cell periphery, similarly to the effect seen in B. D) Huh7 cells transfected with MLN64-Flag and either DIFOPEIN-YFP or YFP alone were subjected to a temperature block at 20°C as described in A. At t = 0, MLN64 exhibits the perinuclear staining pattern that is characteristic of the Golgi. After release from the block, cells transfected with DIFOPEIN-YFP are localized mainly at the plasma membrane, with a small population being localized to vesicular compartments (t = 30). In contrast, cells transfected with YFP alone localize mainly in vesicular compartments, with a small population located at the plasma membrane.

To further characterize the effect of the 14-3-3 mutation on MLN64 trafficking, cells expressing Wt or mutant MLN64 protein were subjected to a 20°C temperature block in order to cause an accumulation of the protein at the Golgi [16]. Cells were then released from the temperature block and MLN64 trafficking was examined by analysis of GFP fluorescence at 0, 15 and 30 minutes after release from the block (Figure 7A).

As expected, both Wt and mutant MLN64 accumulated in the Golgi after the temperature block (Figure 7A, 0 min). However, at 15 minutes after release from the block, only Wt MLN64 protein could be detected in vesicular compartments and the plasma membrane, in contrast to the mutant, which remained primarily in the Golgi complex (Figure 7A, 15 min). Only a small population of mutant protein was seen in endocytic vesicles after 30 minutes, compared with almost all of the Wt protein (Figure 7A, 30 min). That some of the mutant protein does eventually reach endocytic organelles suggests that the effect of the mutation is to delay delivery of the protein to the late endosome after release from temperature block.

The effect of 14-3-3 binding on MLN64 trafficking was also probed by expression of the 14-3-3 antagonist R18 peptide, which when bound to 14-3-3 blocks ligand interactions [17]. This construct, named DIFOPEIN (dimeric fourteen-three-three peptide inhibitor), is a YFP fusion of two tandem R18 coding sequences separated by a short peptide linker [18]. Twenty-four hours following transfection with DIFOPEIN-YFP, cells were probed with an anti-MLN64 antibody to detect the endogenous protein. MLN64 localized to vesicular structures in both DIFOPEIN-YFP-expressing and YFP-expressing cells (Figure 7B, red). However, while MLN64-positive vesicles retained a characteristic perinuclear morphology in cells expressing YFP alone, they seemed to disperse toward the periphery of the cell in cells expressing DIFOPEIN-YFP (Figure 7B). Similarly, when Flag-tagged MLN64 was co-expressed with DIFOPEIN-YFP, MLN64-positive organelles were localized to the cell periphery (Figure 7C). These results further indicate that 14-3-3 binding is involved in proper trafficking of MLN64 to endocytic vesicles.

The effect of DIFOPEIN on MLN64 trafficking was further examined by subjecting cells transfected with Wt MLN64 and DIFOPEIN-YFP to a temperature block at 20°C as described above. Incubation at 20°C caused accumulation of the protein in the Golgi in cells co-expressing DIFOPEIN-YFP and MLN64 as well as in cells co-expressing YFP alone and MLN64 (Figure 7D, t = 0). In cells co-expressing YFP and MLN64, a vesicular pattern of MLN64 expression was detected at 30 minutes after release from the block (Figure 7D). This pattern is similar to that observed with Wt MLN64 alone (Figure 7C). In cells co-expressing DIFOPEIN-YFP with MLN64, MLN64 was still predominantly localized to the plasma membrane, with only a small population found in vesicular structures (Figure 7D), lending further support that loss of 14-3-3 binding causes the trafficking delay observed in the mutant MLN64.

## Discussion

The mechanisms by which cholesterol is exported from late endosomes are not yet clear, but MN64 is implicated in this process due to several observations. The C-terminal START domain of MLN64 has the ability to mobilize cholesterol transport to steroidogenic mitochondria *in vitro* [8] while the N-terminal transmembrane domain has also been shown to bind cholesterol [4]. Although the precise function of MLN64 is still unknown, overexpression of MLN64 has recently been shown to induce expression of the cholesterol efflux regulatory protein ABCA1 [19] and to mediate efflux of cholesterol from late endosomes to mitochondria [20].

Overexpression of MLN64 leads to the appearance of enlarged vesicles, an effect that is also seen when the transmembrane N-terminal portion of the protein but not the C-terminal START domain is overexpressed (Figure 1D). This phenomenon is consistent with previous studies [4] and suggests that the N-terminal domain influences endosomal membrane dynamics. Our results (Figure 1C), in agreement with others’ [1], also indicate that the N-terminal domain of MLN64 is responsible for its endosomal localization, since overexpression of a truncated protein consisting of only the START domain results in ER localization. Interestingly, our results also indicate that mutation of the 14-3-3 binding site in the START domain of MLN64 causes accumulation of the protein at the Golgi and delayed arrival at endosomes (Figure 7A), suggesting that the interaction between MLN64 and 14-3-3 can also affect the localization of MLN64 (see below).

Affinity chromatography using the START domain of MLN64 pulled out a number of proteins that potentially interact with MLN64. Because there are 15 mammalian proteins containing START domains, it is possible that the proteins we identified can interact with one or more of the other START domain-containing proteins, which could explain why proteins such as the mitochondrial import inner membrane translocase subunit (Tim8A) were identified using a cytoplasmic START domain.

Here we show that MLN64 can interact with the cytosolic protein 14-3-3. The 14-3-3 proteins are a group of highly conserved, multifunctional proteins that are involved in such diverse processes as cell cycle control, signal transduction, and protein trafficking [21]. The exact functions of the seven 14-3-3 isoforms has not yet been elucidated, so the significance of the apparent preference of MLN64 for the η and τ isoforms, and to a lesser extent the β, γ, and ε isoforms, is not clear.

While 14-3-3 proteins often interact with phosphoserine or phosphothreonine residues in their ligands, they can also interact in a phosphorylation-independent manner with some ligands, including human telomerase [22], the amyloid beta-protein precursor intracellular domain fragment [23], and ExoS, a bi-functional type III cytotoxin produced by *Pseudomonas aeruginosa* [24]. MLN64 does not contain the conventional binding motifs that are present in most 14-3-3 binding proteins [25] but we have identified a non-canonical binding site in the START domain that mediates the interaction between MLN64 and 14-3-3, which our studies indicate is not dependent on phosphorylation (data not shown). When this site is mutated, the resulting protein interacts much less strongly to 14-3-3 in comparison to the Wt protein (Figure 5C). Furthermore, a higher amount of the mutant MLN64 protein accumulates at the plasma membrane in comparison to the Wt protein, which is predominantly located in endocytic vesicles (Figure 6A); temperature block studies indicate that this phenomenon occurs because trafficking of the mutant protein from the plasma membrane to endocytic vesicles is delayed (Figure 7). Taken together, these results suggest that the non-canonical binding site in the START domain is important for the MLN64-14-3-3 interaction and that this interaction affects the trafficking of the MLN64 protein.

Much of the evidence that implicates 14-3-3 proteins in trafficking comes from studies investigating the regulation of the forward transport of signaling proteins and ion channels from the ER to the plasma membrane [26], [27], [28]. There have also been observations that 14-3-3 proteins affect the nuclear localization of proteins [22], [29]. Our studies indicate that 14-3-3 proteins are also involved in the trafficking of proteins from the plasma membrane to the E/L system. Co-expression of Wt MLN64 with the 14-3-3 antagonist DIFOPEIN delays MLN64 trafficking to the late endosome (Figure 7D) similarly to the results with the MLN64 binding site mutant alone (Figure 6), suggesting that trafficking of MLN64 to the late endosome via the plasma membrane is enhanced by binding to 14-3-3. The importance of 14-3-3 interactions for the internalization of two other proteins to the E/L system has very recently been described [30], [31].

Because MLN64 appears to reach the late endosome eventually, its localization is likely not entirely dependent on interaction with 14-3-3. Instead, it may be that the interaction with 14-3-3 enhances MLN64 endosomal localization by inhibiting its interaction with another protein that would keep it tethered to the plasma membrane. The act of blocking access to other protein partners has emerged as a common mechanism by which 14-3-3 proteins mediate their effects on protein trafficking. Through this mechanism, 14-3-3 can effect the forward transport of ion channels [32], which contain retention motifs that bind to cytosolic COPI (coatamer I)-coated vesicles for retrograde transport to the ER [26]. Properly assembled proteins must overcome this ER quality control mechanism to reach the plasma membrane. Binding of 14-3-3 proteins to “exit” motifs on these proteins prevents COPI binding and promotes protein movement to the cell surface. This mechanism of 14-3-3 masking has been demonstrated for the ER exit-motifs on potassium channels, an acetylcholine receptor and an immune system complex [28]. Similarly, 14-3-3 enhances the nuclear localization of telomerase by inhibiting its interactions with exportin-1, which mediates its nuclear export [22].

In the absence of 14-3-3 binding, MLN64-positive organelles disperse to the cell periphery (Figure 7B and 7C). Endosomal dispersion has been described in cells treated with the actin-depolymerizing drug cytochalasin and in cells depleted of LIM kinase [33], which is involved in actin-mediated dynamics. Endosomal dispersion has also been described in cells depleted of MLN64 by siRNA and was attributed to loss of MLN64-mediated interactions between the late-endosomal membrane and the actin cytoskeleton [5]. However, to date no direct connection between actin and MLN64 has been demonstrated. Our observations that DIFOPEIN expression results in endosomal dispersal (Figure 7B and C) similar to pharmacologic actin disruption and MLN64 depletion indicate that 14-3-3 interactions with MLN64 may mediate its effects on the actin cytoskeleton. 14-3-3 itself is implicated in actin cytoskeleton dynamics by binding and stabilizing the actin-depolymerizing factor cofilin [34]. Taken together, these results point toward a role for 14-3-3 in MLN64-mediated actin cytoskeleton dynamics. Future work should delineate the specifics of the interplay between MLN64, 14-3-3, and the actin cytoskeleton and also determine whether 14-3-3 may interact with other START domain-containing proteins.

## Materials and Methods

### Materials

All chemicals and M2 Flag antibody were from Sigma-Aldrich (St. Louis, MO) unless otherwise noted. All other antibodies, goat serum, and protein A/G beads were from Santa Cruz Biotechnology (Santa Cruz, CA, U.S.A.). Filipin was from PolySciences, Inc. (Warrington, PA). Affigel 10 beads were from Bio-Rad (Hercules, CA). Paraformaldehyde was from Electron Microscopy Sciences (Hatfield, PA). LIpofectamine Plus and Alexa Fluor 594 cholera toxin subunit B were from Invitrogen (Carlsbad, CA). Fugene 6 was from Roche (Indianapolis, IN).

### Cell Lines and Tissue Culture

COS7 and MCF7 cells were cultured in Dulbecco’s modified Eagle’s medium (DMEM), CHO-K1 cells were cultured in 50% HAM’s/50% DMEM and Huh7 cells were cultured in RPMI. All media were supplemented with 10% fetal bovine serum, 2 mM L-glutamine, and 50 µg/ml gentamicin for cell propagation. Cells were maintained in a humidified incubator at 37°C and 5% CO_2_. COS7 cells were transfected with Lipofectamine Plus reagent, and Huh7 cells were transfected with Fugene6 (Roche).

### Cloning

Full length MLN64 (GenBank accession no. X80198) was PCR-amplified from a human liver cDNA library (Clontech, Mountain View, CA) and cloned in frame with a 3xFlag tag into the pIRES-hrGFP-1a expression vector (Stratagene, La Jolla, CA) (MLN64-Flag). To visualize proteins without GFP expression, the GFP coding sequences were excised out of the vector. For the transmembrane (TM) region, nucleotides corresponding to amino acids 1–171 were used, and for the START domain, nucleotides corresponding to amino acids 172–445 were used.

To generate an MLN64 protein mutant (MLN64*-Flag) deficient in 14-3-3 binding, a mutation was introduced at one 14-3-3 potential binding site in the START domain of MLN64: the amino acid sequence KSASN at positions 392–396 was replaced with 5 alanine residues using the megaprimer method [35].

To generate the CD63-YFP fusion, human CD63 (ATCC; IMAGE clone 2819554) was cloned into EYFP-C1 (Clontech) in-frame with YFP. The eYFP was rendered monomeric by introducing the mutation L221K [36].

### Antibody Generation

MLN64 polyclonal antibodies were produced against the START domain of MLN64, spanning nucleotides 526–1335 from the start codon (amino acids 176–445). The fragment was isolated by PCR from a human liver cDNA library using the primers 5′-GCGGATCCCAGGAAGCTGAAGAGGAGCGA TGGTATCT-3′ and 5′-GCGCTCGAGCGCCCG GGCCCCCAGCT-3′ and cloned into the bacterial expression vector pET29a-TRX [37]. The resulting product was sequence verified and expressed in BL21 pLysS bacteria as described [38]. The MLN64 fragment-His6 fusion protein was purified on a nickel-affinity column under denaturing conditions and resolved by SDS/12% PAGE. The protein band was excised and used to immunize New Zealand white rabbits.

### Affinity Purification and Identification of MLN64-interacting Proteins

The bacterially expressed START domain protein was used to generate an affinity column by immobilization to AffiGel 10 beads according to the manufacturer’s recommendations. MCF7 cell lysates were prepared by resuspending the cell pellet in ice-cold MLN64 Lysis Buffer (10 mM Tris pH 8, 150 mM NaCl, 1 mM CaCl_2_, 1 mM MgCl_2_, 1% Triton-X 100) with protease inhibitor cocktail solution (1 µl/20 mg protein). The cell suspension was incubated for 30 min on ice and then cleared by centrifugation at 10,000 g for 15 min and applied to the column. Fractions were eluted from the column with increasing NaCl concentrations and resolved by SDS/PAGE electrophoresis.

For the blot overlay, the 400 mM NaCl elution fraction was resolved by SDS/PAGE, transferred to nitrocellulose and overlaid with recombinant START protein. The membrane was then probed with anti-MLN64 antibody and secondary anti-rabbit antibodies conjugated with horseradish peroxidase. Proteins were visualized by chemiluminescence using SuperSignal West Dura substrate (Thermo Scientific, Rockford, IL).

### Mass Spectrometric Analysis

Protein bands of interest separated by SDS/PAGE were excised from the gel, destained, and reduced with 10mM TCEP, alkylated by 50 mM iodoacetamide, followed by digestion with trypsin (100 ng per band in 50 mM ammonium bicarbonate (ABC)). The tryptic peptides were extracted by using POROS 20 R2 beads (Applied Biosystems) in the presence of 5% formic acid and 0.2% trifluoracetic acid and dried in a vacuum concentrator. The resulting peptides were dissolved in 3–8 ml of HPLC sample solvents containing water:methanol:acetic acid:TFA (70:30:0.5:0.01, vol/vol/vol/vol). Micro-HPLC-MS/MS analysis was conducted on an LCQ electrospray ionization ion trap mass spectrometer (ThermoFinnigan) coupled with an online MicroPro-HPLC system (Eldex Laboratories). Peptides were sprayed directly into the LCQ mass spectrometer (3.6 kV) and data was collected and analyzed using the program KNEXUS (Genomic Solutions).

### Protein Methods

For the glutathione-S-transferase (GST) pull-down assay, GST-tagged 14-3-3 constructs were kindly provided by Peter Mundel (Mount Sinai School of Medicine). The GST proteins were expressed in bacteria, purified using glutathione-coupled agarose, and used in immunoprecipitation assays as described previously [39].

For protein co-immunoprecipitation, cell lysates were prepared in 1 ml of cold co-IP buffer (50 mM Tris pH8, 100 mM NaCl, 5 mM EDTA, 1% Triton X, 0.5% deoxycholate, 10% glycerol) and centrifuged at 10,000 g for 15 min at 4°C to precipitate insoluble debris. Lysates were diluted to 1 µg/µl with cold co-IP buffer and were pre-cleared by incubation with 50 µl protein A/G beads at 4°C for 30 minutes with rocking, followed by centrifugation at 2,000 g for 10 min at 4°C. The precleared lysates were incubated with 2 µg antibody for 16 hr at 4°C on a rocker, followed by incubation with 100 µl protein A/G beads for 2 hours at 4°C on a rocker. The beads were collected by centrifugation at 2,000 g for 10 s and then gently washed 5×5 minutes with 1 ml of cold PBS in IP wash buffer (20 mM Tris pH 8, 1 mM EDTA, 100 mM NaCl, 0.5% Triton-X 100). Beads were resuspended in 100 µl of 5X Laemmli Buffer, heated at 70°C for 10 minutes, centrifuged and used for SDS/PAGE electrophoresis and Western blot analysis as previously described [40].

### Immunocytochemistry

Cells were grown on glass coverslips to 60–80% confluency and transfected according to the manufacturer’s recommendations. At 24 hours post transfection, cells were fixed with 4% paraformaldehyde in PBS for 30 min and permeabilized with 0.1% Triton-X 100 in PBS/10% normal goat serum (NGS) for 30 min followed by incubation with primary antibodies for 1 hour at room temperature. Antibodies were diluted in PBS supplemented with 0.05% Triton-X 100 and 10% NGS. Following incubation with primary antibodies, cells were washed 3×5 minutes in PBS containing 0.05% Triton-X 100. Cells were then incubated with secondary antibodies that were diluted 1:1000 in PBS supplemented with 0.05% Triton-X 100 and 10% NGS for 40 min at room temperature and then 3×5 minutes in PBS with 0.05% Triton-X 100. After a final wash for 5 minutes in PBS, coverslips were mounted onto microscope slides using Fluoromount-G (SouthernBiotech, Birmingham, AL) and analyzed on a Nikon fluorescence microscope fitted with a CCD (charge-coupled-device) camera. Images were acquired with the MetaVue software package and then deconvoluted using AutoDeblur software from AutoQuant Imaging.

For labeling with filipin and antibodies, cells were permeabilized with 0.2% saponin in PBS/10% NGS for 30 min. Primary and secondary antibodies were diluted in PBS supplemented with 50 µg/ml filipin, 0.05% saponin, and 10% NGS. Cells were washed 3×5 minutes in PBS containing 0.05% saponin and then mounted and analyzed as described above.

For temperature block studies, transfected cells were placed in a 20°C incubator for 2 hr, after which they were returned to a 37°C incubator for the indicated times before processing.

### Cholesterol Esterification

Cholesterol esterification assays were performed essentially as previously described [41]. LDL-specific cholesterol esterification was determined by subtracting esterification rates from LDL-plus vs. LDL-minus cells [42].
